# Women’s motivations for choosing a high risk birth setting against medical advice in the Netherlands: a qualitative analysis

**DOI:** 10.1186/s12884-017-1621-0

**Published:** 2017-12-16

**Authors:** Martine Hollander, Esteriek de Miranda, Jeroen van Dillen, Irene de Graaf, Frank Vandenbussche, Lianne Holten

**Affiliations:** 1 Department of Obstetrics, Radboud University Medical Center, Brouwketel 4, 6681 GT Bemmel, Nijmegen, the Netherlands; 2 Department of Obstetrics, Academic Medical Center, Amsterdam, the Netherlands; 3 Department of Obstetrics, Radboud University Medical Center, Nijmegen, the Netherlands; 4 Department of Obstetrics, Academic Medical Center, Amsterdam, the Netherlands; 5 Department of Obstetrics, Radboud University Medical Center, Nijmegen, the Netherlands; 6 AVAG school of midwifery and VU/EMGO research institute, Amsterdam, the Netherlands

**Keywords:** Unassisted childbirth, Home birth, Risk, Freebirth, Care provison, Patient satisfaction, Midwives, Obstetricians

## Abstract

**Background:**

Home births in high risk pregnancies and unassisted childbirth seem to be increasing in the Netherlands. Until now there were no qualitative data on women’s motivations for these choices in the Dutch maternity care system where integrated midwifery care and home birth are regular options in low risk pregnancies. We aimed to examine women’s motivations for birthing outside the system in order to provide medical professionals with insight and recommendations regarding their interactions with women who have birth wishes that go against medical advice.

**Methods:**

An exploratory qualitative research design with a constructivist approach and a grounded theory method were used. In-depth interviews were performed with 28 women on their motivations for going against medical advice in choosing a high risk childbirth setting. Open, axial and selective coding of the interview data was done in order to generate themes. A focus group was held for a member check of the findings.

**Results:**

Four main themes were found: 1) Discrepancy in the definition of superior knowledge, 2) Need for autonomy and trust in the birth process, 3) Conflict during negotiation of the birth plan, and 4) Search for different care. One overarching theme emerged that covered all other themes: Fear. This theme refers both to the participants’ fear (of interventions and negative consequences of their choices) and to the providers’ fear (of a bad outcome). Where for some women it was a positive choice, for the majority of women in this study the choice for a home birth in a high risk pregnancy or an unassisted childbirth was a negative one. Negative choices were due to previous or current negative experiences with maternity care and/or conflict surrounding the birth plan.

**Conclusions:**

The main goal of working with women whose birthing choices do not align with medical advice should not be to coerce them into the framework of protocols and guidelines but to prevent negative choices.

Recommendations for maternity caregivers can be summarized as: 1) Rethink risk discourse, 2) Respect a woman’s trust in the birth process and her autonomous choice, 3) Have a flexible approach to negotiating the birth plan using the model of shared decision making, 4) Be aware of alternative delivery care providers and other sources of information used by women, and 5) Provide maternity care without spreading or using fear.

## Background

The Netherlands are often praised by natural childbirth advocates and heralded as a haven for physiological, natural (home-) birth. However, in spite of the fact that home birth is still a valid and respected option within the system in the Netherlands, it has been declining in recent decades. Healthy women without a problematic medical or obstetrical history or pregnancy complications can still opt for home birth with a midwife, but more and more women are referred to the hospital either in pregnancy or during birth for an increasing number of indications, including women’s own requests [1]. On the other hand, many Dutch obstetricians and midwives have the impression that a growing number of women refuse to be referred. They choose home birth against medical advice, with or even without a midwife present. This has become a ‘trending topic’ of many conferences and symposia in the Netherlands in the last 3 years. However there is, as yet, no statistical data to support this impression. These choices can give rise to legal and ethical dilemmas, as described in several publications [2–4].

In a recent scoping review, Holten and de Miranda found 15 studies on the motivations of women choosing unassisted childbirth (UC), home birth in countries where home birth was not well integrated into the maternity care system, or a midwife-attended high-risk home birth [5]. The countries involved were Australia, Canada, Finland, Sweden, the United Kingdom (UK) and the United States of America (USA). Women in these studies who chose to give birth ‘outside the system’ often put their trust in their own intuition, thereby resisting the biomedical model of birth and challenging the dominant risk discourse by considering the hospital as a dangerous place. These women often perceived birth as an intimate or spiritual experience. They felt that true autonomous choice was only possible at home. For some women in these studies, taking full responsibility for the birth outcome (good or bad) was a reflection of true control over decision-making. The key conclusion of this review was that ‘concerns over consent, intervention and loss of the birthing experience might be driving women away from formal healthcare and that there is a lack of fit between the health needs of some pregnant women and the current system of maternity care in several high-income countries’ (p.55). Furthermore, the authors argue that a dialogue on views on superior knowledge, risk, autonomy and responsibility should take place between women and their health care providers.

Also recently, two similar studies from the UK reported that women often feel that their rights are violated: UC is legal, but not always treated as such by professionals [6, 7]. Therefore they have to plan tactically and keep their intentions a secret. They believe they are judged by social services to be unfit as a mother. The authors also found that participants objected to professionals only talking in risks, and felt subjected to a system dominated by fear of a bad outcome.

It is tempting to attribute the choice that women make for home birth in a high risk pregnancy or UC in the countries mentioned above to a lack of physiological approach to childbirth and high percentage of interventions. However, in the Dutch system the same phenomenon is seen, even though midwifery care and home birth for low risk women are integrated in the maternity care system and rates of interventions (for instance induction of labor, use of analgesia and caesarean section) are still relatively low. This is despite an increase of referrals from primary to secondary care in the last decade [8]. Therefore it is necessary to look beyond increasing medicalization and access to home birth, and examine Dutch women’s motivations and their negotiation with medical professionals in maternity care to elucidate this issue.

To this end the WONDER-study (Why women want Other or No DElivery caRe) was conceived. We used a mixed methods study to explore the motivations of Dutch women who have chosen to give birth ‘outside the system’ (e.g. against medical advice and/or guideline/protocol or UC) and the experiences of midwives and obstetricians regarding care for these women. In this paper we present the results of in-depth semi-structured interviews with 28 women on their motivations for choosing home birth in a high risk pregnancy or UC and their approach to realise the intended birth of their choice.

## Methods

For the purpose of fully reporting the process of data collection and analysis of the findings, the COREQ criteria were used [9]. For this study, permission was sought from the medical ethics committees of the Radboud University Medical Center Nijmegen and the Academic Medical Center in Amsterdam. Both deemed the study as not requiring permission.

### Research team

All interviews were conducted by one of three authors (MH, LH and EdM), who are also women and researchers with a professional interest in women’s motivations to give birth outside the guidelines. All have a medical background in midwifery/obstetrics and had experience with in depth interviews. One (LH) had extensive previous experience with qualitative research as a medical anthropologist. Prior to the interviews, none of the subjects were known to the interviewers, either personally or professionally. However, there had been email contact with all participants, asking for their participation and explaining the reasons, goals and methods of the study and the identity and background of the interviewer.

### Study design

**Fig. 1:**
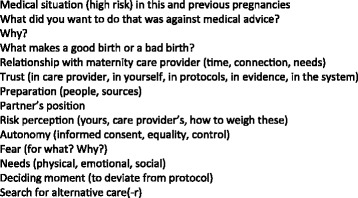
Topic List. List of topics used during the interviews

This study consists of exploratory qualitative research using a constructivist approach and a grounded theory method [10]. Participants were selected through several sampling methods: purposive (approaching certain nationally known advocates or famous “cases”), convenience (contacting potential participants who happened to be posting on an online maternity care users forum during the time of recruitment) and snowball (referral of some participants by other participants or their midwives, who were informed about the study by the researchers). The sole criterium for inclusion was one or more births “outside the system”. Before the start of the study consideration was given to the question whether women who had a UC and women who had a midwife who attended their high risk home birth should be analyzed in the same study. Halfway during the interviews it became clear that the motivations and perspective of women in both groups were very similar. Therefore the decision was made to include all of the participants in this study. All participants were approached by online methods. There were no refusals or drop-outs. All participants gave informed consent for their quotes to be used in this article. All but one of the interviews took place at the home of the participants. One interview was held in the Academic Medical Center in Amsterdam by the participants’ request, for logistic reasons. Demographic data of the participants are shown in Table 1. The interviews were semi-structured by the use of a topic list [Fig. 1], which was based on themes known from the literature [5] and questions the researchers had themselves, though the interviews were allowed to flow naturally. Certain topics (e.g. defining moment, search for alternative care) were added later during the study as they had been mentioned by participants in earlier interviews. All interviews were recorded by digital sound recorder and transcribed verbatim either by a commercial company or by volunteer medical students. The interviews lasted between 30 and 120 min, and some field notes about atmosphere and personal observations were made afterwards. All sound files, transcripts and informed consent forms were stored anonymously in a secured password protected university digital storage system.

**Table 1:** Maternal characteristics (*N* = 28, involving 35 deliveries)

Maternal characteristics	N
Indication for secondary care	
VBAC (1 also diabetes type I)	21
Breech (1 also post term)	8
Twins (1 also preterm)	5
Previous postpartum hemorrhage (>1000 ml) or	3
manual placenta removal	2
Prelabor rupture of membranes > 24 h	1
High body mass index (> 35)	1
Treatment with low molecular weight heparin	1
Unassisted childbirth (UC)	7
Age at delivery (years)	
20-25	2
> 25-30	18
> 30-35	8
> 35-40	7
Parity during relevant delivery	
1	8
2	13
3	8
4	4
5	1
6	1
Employed	
Yes	19
No	9
Highest education	
High School	4
Vocational training	4
College	6
University	14
Marital status at time of relevant delivery	
Married	20
Living together	8
Perinatal death	2
Breech	1
VBAC	1

### Data analysis

**Fig. 2:**
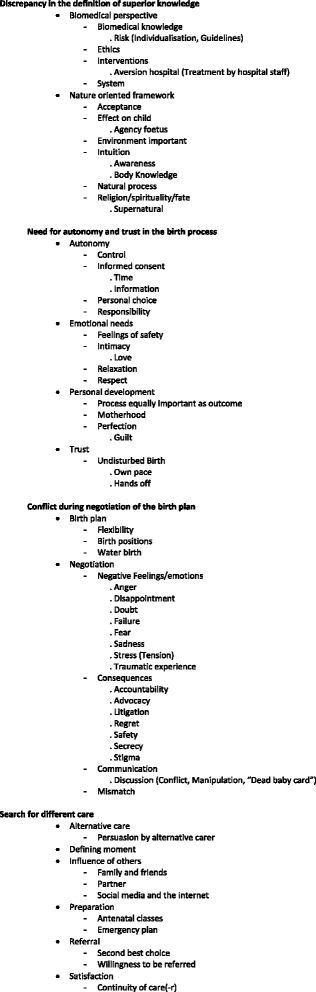
Code Tree

Data were analyzed by two of the authors (LH and MH) using qualitative data analysis software (MaxQDA). Before the start of coding and twice during the coding process (after approximately ten and twenty interviews) LH and MH coded the same interview and discussed the differences in coding in order to check intercoder reliability and reach consensus. The coding was started bottom up and expanded and built on during each additional interview. After approximately ten interviews an interim thematic analysis was done, and themes from this were then incorporated into the topic list for subsequent interviews. Data saturation was reached after analysis of the first 22 interviews; analysis of a further six interviews confirmed this. The final coding tree [Fig. 2] was decided on by consensus between LH and MH after all coding had been completed. Transcripts were not returned to the participants. Instead, to validate and discuss the themes that were found, a feedback focus group was held. Six participants were purposefully selected from the 14 who were willing to participate in this because of their different stories and obstetric histories. Two of the authors (MH and LH) translated the quotes that are used from Dutch to English.

## Results

Twenty-eight women were interviewed. After grounded theory analysis of all interviews four major themes emerged: “Discrepancy in the definition of superior knowledge”, “Need for autonomy and trust in the birth process”, “Conflict during negotiation of the birth plan” and “Search for different care”. After careful consideration of the data it became clear that one overarching core category connected the four major themes and all their sub-themes, and this was fear.

### Discrepancy in the definition of superior knowledge

All participants described a discrepancy between the views of their regular maternity care providers and their own views regarding risk perception of childbirth. In their experience, the professionals’ starting point was a biomedical framework based on protocols and guidelines, in which (screening for) risk factors and using interventions to minimize risk was the mainstay of their approach. The participants had differing views on childbirth which can roughly be divided into two schools of thought. One group used a traditional biomedical framework, but weighed risks and benefits differently from their providers. For instance, they believed that the negative consequences of the suggested procedures far outweighed the possible prevention of harm. Because of this, they were prepared to accept the small increase in risk that refusing certain interventions entailed. This group questioned the applicability of the evidence used by medical professionals because they believed that the optimal way to approach childbirth (for instance a breech on all fours) had never been tested against the current standard. Also, these participants believed guidelines and statistics applied to large groups but have very limited use for the individual woman.

*“I found the risk of a uterine rupture of 0.4% acceptable. Because other than that I had absolutely nothing. […] [Weighed] against the risk of intervention or even another caesarean in the hospital. […] so I came to a 0.4 for me, individually” (R18, home VBAC (vaginal birth after cesarean), second child)*

The other group used a nature-oriented framework, wherein a pregnant woman’s intuition is considered superior knowledge. They deemed childbirth only safe if left alone and taking place undisturbed in an atmosphere of relaxation (usually at home), where the woman can follow her intuition. However, giving birth in the stressful environment of a hospital, or even at home in the presence of a midwife, would lead to more interventions, making the situation less safe.

*“I believe that I could get some of the same answers with my intuition, that you could measure in the hospital with machines. […] Your own consciousness could also give you signals, a sense of what needs to happen next” (R1, home breech birth, first child)*

Several participants also believed that the way a child was born and the atmosphere it was born into would have an effect on its development in later life.

*“I think many UC women believe, I know I do, that many problems growing up and being human (…) are rooted in how we are born. (…) When I look at society and how harsh and cold it has become and how individual, I think: yes, I am not surprised when you see how we are all born. I see a connection there. “ (R6, UC, second and third child)*

### Need for autonomy and trust in the birth process

Many participants expressed a strong need for autonomy during labor and delivery. They stated that, in their experience, midwives and obstetricians often did not ask for consent before performing invasive procedures (for example episiotomies, rupturing membranes, performing an assisted vaginal delivery or even a cesarean section). Many were traumatized by this during a previous delivery, which contributed to their decision to reject medical advice this time.

*[…] “And he rammed that vacuum pump in, literally. Like that! He said: ‘I am not here for my own amusement, I am here to help you.’ And he rammed […] that vacuum pump in without consultation [with me]. […] And then it was a C section. […] And I think it is mostly because I had that C section. […] and if there would not have been that last traumatic part that doctor X [gynaecologist] came in...[...] then I don’t think that I would have necessarily ended up here [giving birth at home].” (R18, home VBAC, second child)*

Participants also mentioned the need to feel safe, loved and respected during their delivery, and be surrounded by people who trusted in their ability to give birth unaided, which they felt would not be possible in the regular system.

*“[My midwife] wanted to know what was going on and she wanted to perform examinations, and I knew for sure that she would not be ‘hands off’. That was stressful for me and I became nervous every time I thought about it. […] I didn’t want someone who wanted to examine me and did not trust me and therefore I couldn’t trust my body and I would produce stress hormones.” (R2, home breech birth, first child)*

For some, the process of an undisturbed natural birth was (almost) equally as important as the outcome, as it was part of the personal development of the mother in becoming who she wanted to be: an autonomous woman without fear. Most participants believed that a birth without interventions would be more likely to lead to the desired outcome of an (emotionally and physically) healthy mother and baby.

*“It can be so affirming, a delivery. It is such a lifelong effect, your experience. […] And yes, I have really become a different person through that delivery because I really faced all my fears. Because I really did it myself and it wasn’t the midwife who ‘did’ my birth.” (R3, UC, third child)*

Some participants who had chosen a UC indicated that, in their experience, health care providers believed they were responsible for the outcome of a delivery, whereas the participants themselves insisted that true autonomy was only possible when they were allowed to take full responsibility for their own decisions and whatever outcome that would lead to.

*“I am the woman who is giving birth, so I am ultimately responsible, even if you are standing next to me, I am still responsible for what I decide to do.[…]” (R5, UC, fourth, fifth and sixth children)*

When discussing her midwives’ reaction to her intention to give birth unassisted, the same participant later said:

*[…]“Their fear reaction was: ‘Yes, but then we are responsible for something we are not present for.’ Which I felt did not make sense, because you are not there, so you can’t be responsible either. But they were very afraid of repercussions if things went wrong, or that we would hold them accountable […].” (R5, UC, fourth, fifth and sixth children)*

Noticeably, none of the participants regretted their choice to birth outside the system, not even the two whose baby did not survive.

*“For me it feels very clear […]. That now my conscience does not bother me and that I can imagine that would be more the case if I had not been able to make my own decisions surrounding the birth.” (R28, home breech perinatal death, first child)*

### Conflict during negotiation of the birth plan

Most of the participants started in regular care during the index pregnancy, by which we mean the first pregnancy in which they chose to deliver “outside the system. For many, the decision to give birth at home (or unattended) against medical advice was made sometime over the course of the pregnancy. Many times, one of the deciding factors was a conflict over (part of) the birth plan. Items that were often grounds for discussion were birth positions or the desire for a water birth in a high risk pregnancy. As one participant, who experienced a uterine rupture during a home VBAC, said:

*“And [the gynecologist] said to me, ‘I can’t offer you that bath’, but if she had, I think that would have convinced me to choose the hospital. And it may be stupid to say, was it really just that water birth, that made you take all those risks […]? Yes, I did that. […] We did not take that decision lightly. […] An instinctive knowing that that is the way I could give birth AND that it was denied me last time and I let that happen.” (R23, attempted home VBAC, second child)*

Another participant, who experienced a perinatal death during a home breech delivery, stated:

*“In the hospital it was very likely that I would have to give birth lying on a bed, I was afraid of that too. [...] and I felt a very strong fear: if I had to lie down I would not be able to get him out. I had to be able to move around. [...] In our experience we were not impossible to talk to about this subject...[...] no.” (R28, home breech, perinatal death, first child)*

Also, some participants desired to waive certain parts of the protocol, for example continuous CTG (cardio-tocography) monitoring during VBAC. Most participants felt they encountered insufficient flexibility on the part of their provider. According to them, discussions about the birth plan often involved manipulation on the part of their providers, including threats of perinatal mortality if protocol was not followed. This has become known amongst many participants as ‘playing the dead baby card’, also known as ‘shroud waving’ in English literature [7].

*“In between I had an unpleasant consultation, [the obstetrician said] ‘Yes, at 41 weeks it will be a C-section (cesarean section).’ I thought: why? And: ‘You don’t want a dead child and that we will end up across from each other in court?’ So within five minutes we had a grim discussion.” (R15, home VBAC, second child)*

This negotiation then led to feelings of anger, disappointment and stress on the part of the participants. In quite a few cases, the decision to go against medical advice had negative consequences for the women involved. Some had child protective services forced on them, and many felt they had to operate in secret because of this and the stigma it involved.

Only two of the 28 women did not encounter conflict in the negotiation of their birth plan. One involved a positive first choice for UC with full (stand-by) support of a midwife. The second woman started care with an alternative midwife, possibly without realizing that this midwife did not adhere to national protocols.

### Search for different care

Many times, there was a “defining moment” (often a conflict or major disagreement) during antenatal discussions with an obstetrician or midwife, which led to the participants’ decision to give birth elsewhere, alone, or with another (often “holistic”) midwife. Frequently, this midwife would be a single practitioner, often specializing in working outside the guidelines, and providing continuity of care both during pregnancy and delivery. Many participants indicated that they experienced a lack of continuity of care (−r) in regular care. Some were under the care of a group midwifery practice, with anywhere from four to a dozen midwives. Others were cared for in a hospital, and saw many different obstetricians, residents and clinical midwives during the course of their pregnancy. Some had started a previous birth in primary (midwifery-led) care, but were transferred during birth to a clinical setting due to a complication, at which point their own midwife left them in the hands of hospital staff they had never met before.

Often, women turned to social media, for instance certain facebook groups like “the birth movement”, to find confirmation and inspiration for their choices, and to connect with a “holistic” midwife. In some cases, communicating with like-minded individuals and providers through the internet confirmed women in their decision to make a different choice, whereas in other cases their minds had already been made up.

*“[After finding out the baby was breech] And then I cried in the car. [...] And then I thought: yes, now it won’t be a home birth any more. […] Then I cried for I think another hour. Then I went on the internet and joined the birth movement […]. And then within an hour I had somebody who said: ‘I will help you at home together with your [own] midwife.’”(R17, home breech birth, first child)*

*“ I was about 34 weeks I think and then I joined the Free Birth Group on Facebook and there was [midwife] too. [A friend] said: [midwife] is first-rate. I could say whatever I wanted and she would do it. So I called [midwife].” (R20, home VBAC, second child)*

Some participants quickly found a likeminded new caregiver, others searched for quite some time and experienced rejection (of their wishes) by yet another midwife or obstetrician.

*“[…] I had really called or approached every [midwifery] practice in [the city] and they all had the same story, so I felt like either you all have that same protocol that you follow to the letter, OR you have discussed me [between yourselves], but I noticed I could not get a foot in the door.” (R11, high BMI (body mass index), home birth second child)*

Some participants proceeded with their pregnancy without medical help. They checked their own blood pressure, measured their own abdominal circumference, or had an ultrasound done to check for placental location. Some of those who planned a UC devised emergency plans for the most common critical situations, like shoulder dystocia or post partum haemorrhage, whereas others notably did not, since they trusted that an uninterfered-with birth would not go awry.

*“[…] I had instructed my partner that if I...suppose I were to lose a lot of blood, really a great deal of blood....the bath fills quickly but you can certainly see the difference....if you couldn’t see my legs any more [...]. But mostly that he had to pay attention to me. If I seemed somewhat distracted or sleepy, that he had to call [the alarm number].”(R5, UC, fourth, fifth and sixth children)*

Many of the participants spent a significant amount of time preparing for birth. They read books, took antenatal classes (often hypnobirthing) and talked with family and friends about their decision. Although every participant discussed her situation with her partner at length, it is noticeable that most stated that their partner left the search for information and the final decision completely up to her.

### Fear

Women felt that their care provider’s version of superior knowledge, with its evidence based protocols, stemmed from fear. Most participants believed that an optimal birth could only be achieved through true autonomy and trust in the natural process, and that this was only possible without fear. According to the many of the participants, conflicts often arose because of fear: where health care providers were afraid of a bad outcome or litigation (or both), women feared unnecessary interventions, being overruled and losing their autonomy, having their birth disturbed (by interventions), being reported to social services and being stigmatized for their choices. These conflicts were an important factor in women’s search for a care provider and/or birth setting without fear.

*“That CTG or that doptone is also based on fear. Yes, then you trust the machine more than what I tell you about how it’s going, or your own intuition. And I understand that you think, as a midwife, you don’t want to be sued, and you don’t want a dead child, and you feel responsible. I understand all that. But it takes away my control over my delivery and my body and what I want.” (R6, UC, second and third child)*

The participants in the feedback focus group acknowledged the four main themes as generally fitting with what they had told the interviewers, although several had difficulty with the term fear. They were concerned that fear as an overarching theme would make them appear to be weak and afraid, whereas they viewed themselves as strong, enlightened and determined. The authors therefore emphasize how the theme fear does not just reflect on the participant’s fear of unnecessary interventions, but much more on the medical approach of childbirth at this time, with its fear of bad outcome, peer pressure and legal measures.

## Discussion

This qualitative study involved 28 in-depth interviews with women who made choices for their birth setting that went against medical advice. Four main themes and one overarching theme emerged. These will now be rephrased as positive recommendations and discussed with reference to the literature.

### Rethinking risk discourse

The central concept of the first theme, “Discrepancy in the definition of superior knowledge”, is risk discourse. Emphasis on risk has in recent decades become a dominant aspect of clinical discourse, where obstetricians and midwives use protocols and guidelines to minimize risk of morbidity and mortality for the mother and her developing child. In the Netherlands this became more explicit after the publication of the PERISTAT (perinatal statistics) reports in 2008 and 2013 in which perinatal health indicators of 29 European countries were compared. The perinatal mortality rates of the Netherlands were relatively high in comparison to other high income countries [11]. This is felt by many to have resulted in a stricter use of national guidelines and more local protocols which can be seen as a process of re-evaluation of the boundaries of physiological birth. Scamel and Alaszewki describe this as an ‘ever narrowing window of normality’, in which normality is defined as the absence of risk [12]. Another reason for the current risk discourse can be found in the increased scrutiny in maternity care, where bad outcomes can become subject to reviews, audits and medico-legal consequences. A policy focused on risk reduction, however, frequently leads to an increase in the number of interventions, including induction of labor, cesarean section, episiotomy, fetal heart rate monitoring during physiological birth, even hospital birth itself. All of these interventions naturally come with false positives (for instance “unneccesareans”) (http://www.betterbirthblog.org/breech/cesarean-breech-birth/an-easy-cesarean/). As Bisits puts it: “Most of the risks in maternity care refer to low prevalence phenomena. Prevention or mitigation of these risks usually requires the treatment or management of large numbers of women in order to avoid an adverse outcome. This unavoidably results in over-treatment” ([13] p.13). The focus of risk discourse in maternity care, however, is usually on what numbers of overtreatment are acceptable when prevention of mortality or serious morbidity is at stake.

Some women feel like they are not being adequately counseled on the cost of a proposed intervention for the sake of risk minimization. Instead of numbers needed to treat, numbers needed to harm and exact incidences in percentages, they were informed by means of relative risks or odds ratios, concepts that are abstract and difficult to understand, even for health care providers themselves [14]. These women experienced a clash between differing risk perceptions, prompting some women in the current study to make a negative choice to leave the system. They indicated they needed an alternative for ‘risk talk’. Risk talk as such cannot, and should not be completely avoided due to requirements of informed consent and informed choice, but midwives and other maternity care providers can use different techniques to put risk into perspective. It is important to realize that the way providers talk about risk and the strength of recommendations can be influenced by previous experiences and/or the dominant risk approach (culture) in the health institute of the maternity care professional [15]. Van Wagner suggested that risk talk of professionals can be prone to exaggeration [16]. As Scamel and Alaszewki state: “in midwifery conversation normality has no language of its own and has to be defined against the dominant discourse of high risk” ([12] p.216).

Other women reject medical risk discourse altogether. They trust their instincts, believe that childbirth is a natural process and inherently safe, and locate risk in the interventions of caregivers [7, 17]. These women sometimes make a positive choice to leave the system.

### Respecting a woman’s trust in the birth process and her autonomous choice

The second theme, “Need for autonomy and trust in the birth process”, demonstrates that autonomy is a very important concept for most women who choose to go against medical advice in their birth choices. This is in accordance with previous studies, where this theme is frequently mentioned [18–24]. Autonomy in these studies included deciding how and where to give birth and who can be present at the event, and required full informed consent for every intervention. This even encompassed some minor or routine interventions by professionals, like taking a blood pressure, rupturing the membranes or performing an abdominal examination. If autonomy is overruled, this may lead to a traumatic experience and to women making a negative choice to leave the system. Full autonomy by necessity also means full responsibility. Many professionals believe that, because they have had substantial training and experience, and are authorized to make clinical decisions, they are responsible for not only the process, but also the outcome of a birth, for both mother and baby. However, women who choose to go against medical advice during birth feel that, if they made a fully informed choice, they themselves are ultimately responsible for the consequences of that choice, be they bad or good [24–26]. Some of these women even rejected the term “shared decision making”, a concept that has become the current standard in counselling and informed decision in health care [27]. They felt that only one person could make a decision, and that should be them. However, shared decision making encompasses much more than provider and patient deciding on a course of action together. It also means involving patient preferences, background and culture in every decision on health needs, and has been shown to improve patient satisfaction in birth experience [28].

Most women in the current study expressed the need to be supported during birth by professionals and partners who, like them, trust in the birth process. They believed that if they were surrounded by professionals who saw birth as ‘risky’, that this could prove to be a self-fulfilling prophesy. Those present might, because of their perception of the inherent ‘unsafety’ of childbirth, be tempted to intervene in the natural process, thereby disturbing the flow of the birth and causing the very problems they were trying to prevent. As Wickham stated: “It may be uncomfortable to realise that ‘we’ can also be seen as an intervention, but if we can find ways of listening carefully to what this minority of women are saying we may be able to find ways of improving the experiences of all women” ([29] p.5). Recently Symon et al. published a scoping review of this phenomenon of self-fulfilling prophesy described as the ‘nocebo effect’ [30]. He concluded that “it appears that nocebo is significantly more common in women and where there is prior negative knowledge/expectation (p.1526).”

In summary, it appears that women wish to be supported by someone who views and trusts birth as they do. For some this means: inherently safe if left alone. Also, in order to maximize their chances of an uncomplicated birth, they want to experience complete autonomy in all choices surrounding the birth. Ultimately this may mean also accepting final responsibility for the outcome for both themselves and their baby.

### A flexible approach to negotiating the birth plan using the model of shared decision making

The third theme in the current study was “Conflict during negotiation of the birth plan”. Feeley describes conflicts women experienced *after* making the *positive* choice for UC [6]. Conversely, in the current study, many women made a *negative* choice to leave the system *because* a conflict arose with their provider during the current pregnancy, or a previous one. This conflict frequently concerned their wishes for their birth plan.

Many women who ended up giving birth at home in a high risk pregnancy, or even unattended, started their current or previous pregnancy in regular care. Somewhere along the way a mismatch occurred between their childbirth wishes and the plan suggested by their provider. They experienced little or no shared decision making, but, in contrast, were confronted with “the protocol”, deviation from which they found to be not open to discussion. Providers, on the one hand, have more extensive knowledge of the physiology and pathology of childbirth than most of their clients and use evidence based medicine to decide on a treatment plan. But they can also experience pressure from their institution and their colleagues to adhere to protocols and consider birth to be abnormal until proven otherwise [31–33]. This can appear as defensive medicine to some women. Participants in the current study feared that the policy suggested by their provider would prevent them from having the birth they wanted and would lead to more interventions, which would only worsen the outcome for them and their babies.

Birth plans are relatively new in maternity care. Introduced by childbirth educators in the nineteen-eighties in the United States, they became a way for women to defend themselves against the rising rate of interventions in US hospitals [34]. Jenkinson et al., in Australia, found that, among women who wanted to deviate from standard protocol, those who had a birth plan had more chance of achieving their desired birth [35]. On the other hand, Mei et al. in the United States reported that the number of requests in a birth plan was inversely related to the level of patient satisfaction, unless those requests were honoured [36]. Unfortunately, rather than improving relationships, birth plans may irritate the staff, which adversely affects obstetric outcomes [34]. In other words, patients with birth plans are seen by medical personnel as “difficult”, and almost setting themselves up for disappointment. Debaets et al. reported that many maternity care providers ignore birth plans because they feel they were made thoughtlessly and without prior discussion with the care provider themselves [37]. This made them recommend that patient and provider write the birth plan together, a variation of the concept of shared decision making. This irritation on the part of the caregiver was keenly felt by many participants in the current study, and was thought to have contributed to the conflict. Participants indicated that they experienced very little flexibility in their provider’s attitude, and felt they had no other choice than to give birth elsewhere or with another provider. This is in accordance with a study by Keedle et al., who found that women who gave birth at home after a previous caesarean section did so due to inflexible hospital systems and inflexible attitudes [38]. These women found little or no support for their choice to attempt a VBAC in the regular system, or felt they had a better chance of a successful VBAC at home.

In summary, conflict over the birth plan caused by an experienced lack of flexibility from the provider may lead some women to make the choice to leave the system in the current or following pregnancy. If providers could recognize the “defining moment” and act on this, these negative choices might be prevented. This could perhaps be achieved by an open, empathetic attitude, negotiation using the concept of shared decision making, and an awareness that second best care (in the eyes of the provider) is a better alternative than a home birth for a high risk pregnancy, or no care at all.

### Awareness of alternative delivery care providers and other sources of information

The theme “Search for alternative care” describes the women’s search to find a care provider without fear, who will respect their autonomy, provide continuity of care and share their views on childbirth.

The last decade has yielded several studies -in different settings- on women’s motivations for going against medical advice in their choices of place and provider for their delivery [5–7]. However, not much is known about how these women then proceed. Although most women in the current study decided on their own that they wanted to deviate from the medical protocol their provider had recommended, most did not reach their final decision without any outside influence. They became aware of alternatives to regular care by reading books, often written by natural childbirth advocates like Ina May Gaskin, Laura Shanley or Helene Vadeboncoeur [39–42]. The ideas of these authors were often quoted by the participants. They also took childbirth education classes, most often hypnobirthing. In accordance with the findings of Miller and Feeley, most if not all visited peer support websites dedicated to natural childbirth, unassisted childbirth and home birth in a high risk pregnancy (for instance breech and/or VBAC), where they found information on the options available to them, and access to sympathetic midwives [6, 19].

A perceived lack of continuity in care contributed to the participants’ dissatisfaction and search for an alternative. The holistic midwives that were present at these high risk home births met that need. They performed all antenatal checks personally, and stayed with women who were in labor until the baby was born, even if a transfer to another setting became necessary. Dahlen et al. likewise found that many participants in their qualitative study chose UC because of a lack of continuity in the hospital system [43]. They report that Australian women who opt for home birth or UC also found this continuity in doulas and (lay) midwives. In contrast to these findings, a recent structured literature review found that, in a general population, women wanted consistent care from caregivers that they trusted, but did not value continuity of carer for its own sake [44].

In summary, women in the current study searched for alternative information through books, internet or their social network, and often found a care provider who could deliver continuity of care(−r).

### Maternity care without fear

Fear was the core category that united all themes in this study. Opting for a home birth in a high risk pregnancy or for UC is not an easy path and can be inspired by both positive and negative emotions [17]. Some of the participants in the current study were motivated by positive emotions. These women chose UC or home birth in a high risk pregnancy as a first choice, because, although they did not necessarily object to the presence of a midwife or hospital care in itself, they believed that such care had no added value in their situation. Other important ingredients for an optimal birth experience were an atmosphere of intimacy, relaxation, love and respect surrounding the birthing mother. Many participants felt that birth in such an atmosphere was a necessary requirement in order to become who they needed to be, “an empowered autonomous woman and mother” [24, 45]. Others made a negative choice, where they had previous (or current) unsatisfactory experiences (in health care) and did not want to subject themselves to such care again. They chose a different setting for their delivery in order to avoid the alternative.

As Dahlen wrote recently: “Childbirth is no exception to this temptation to control through fear” ([46] p.8). Several of the participants in the current study mentioned ‘shroud waving’: their provider telling them they were risking the life of their child by making the choice for a home birth in a high risk pregnancy, or a UC. The participants felt this was not only indicative of coercion, but also of their provider’s fear of a bad outcome. The theme ‘provider’s fear’ is also mentioned in literature. Plested et al. interviewed ten women who had a UC in the United Kingdom [7]. They found that the fear of professionals for a bad outcome dominated medical discourse so much that participants felt burdened to the extent of withdrawing from care altogether. Jefford and Jomeen describe the effect of fear of a bad outcome on midwives working in the National Health Service in the UK [31]. They report how the midwives who were interviewed regularly felt they had to disregard their inclination to advocate for the rights of their birthing patients because of institutional policies and fear for their job or position.

Women’s fear of medical professionals’ interventions can also blind them to real risks involved in a UC or home birth in a high risk pregnancy. Dahlen in “Undone by fear? Deluded by trust?” describes two cases [47]. In one, a woman with two previous uncomplicated births died after having an elective caesarean section for a breech position. She was undone by fear. Her counterpart, a vocal Australian UC advocate, died during an unassisted home birth. She was deluded by trust. The author argues that both unmitigated fear (implied: imposed by professionals), as well as unconditional trust in the natural course of childbirth can lead to a bad outcome. This unconditional trust was certainly voiced by some of the participants in the current study. They believed that most if not all medical interventions are unnecessary and will only cause a cascade of further interventions, leading to a bad outcome. For instance, several of the participants believed that shoulder dystocia and post partum haemorrhage do not occur in unassisted childbirth and are always due to providers’ interventions.

In summary, the participants in this study described two dimensions of fear: their own fear of a cascade of unnecessary interventions, and their provider’s fear of a bad outcome and the repercussions thereof.

### Implications for practice: Preventing high risk choices for negative reasons

This study demonstrates that some women choose a home birth in a high risk pregnancy or a UC as a positive first choice, whereas others do so out of negative associations with maternity care. New insights generated by this study highlight the negotiation and conflict surrounding the birth plan, and the search for alternative care. Many caregivers feel frustrated and concerned for both the mother and the baby’s welfare when confronted with a pregnant patient who refuses routine care or even any care at all. They wonder how they can get the patient to comply with medical advice. But perhaps this is the wrong approach in these situations. If the woman’s choice is a positive one, it seems there is little or nothing a caregiver can do or offer that will make her change her mind. However, if the choice is negative, there is a reason why a woman is choosing to avoid certain measures that are offered to her, and we should be asking her why. As this study shows, many women who reject medical advice have been traumatized during a previous birth, where they felt left alone, not taken seriously, or even violated. The women in this study felt that in hindsight certain interventions done to them in the past were unnecessary, or even harmful. They felt they were not properly informed and did not give full informed consent.

If we as health care professionals wish to prevent women from making what we consider high risk choices for negative reasons, there is much to be gained from preventing traumatic experiences. We must face that in daily practice difficult situations can arise when evidence based medical knowledge clashes with women’s views. However, in this time of increasing use of shared decision making and a growing awareness of the importance of patient relevant outcomes such as patient satisfaction with care [48], new ethics are required in maternity care. Equal partnership between care provider and pregnant woman is a prerequisite for a transparent dialogue, where counselling is done without coercion and with full disclosure of all known facts. These facts should be presented as absolute risks, numbers needed to treat and numbers needed to harm, and clear information should be given about what is not actually known. Threats and “shroud waiving” should be avoided and informed consent is required for any and all interventions. Furthermore, an attempt should be made to minimize changes in caregiver, thereby increasing continuity of care. If it becomes clear the woman persists in her high risk choice, she should be told that she will always be welcome in “regular” maternity care if she changes her mind or if complications arise.

There will always be some women who make a positive choice to take a different route, but negative choices are undesirable for both women and providers. The main goal of counselling should not be to bring as many women as possible within the framework of protocols or guidelines, but to prevent negative choices.

### Strengths and limitations

All authors are or were involved in maternity care and are committed to the improvement of birth outcomes. The interviewers were familiar with the Dutch maternity system. This background is visible in the topic list and the importance assigned to the results regarding women’s autonomy, although autonomy is an important theme in all the international literature on this subject. Participants were aware that interviewers, as medical professionals, were (formerly) part of the ‘system’ which they critiqued. It is possible that for some this led to a certain reticence in answering freely. On the other hand, the medical background of the interviewers made it possible to quickly discern which questions were relevant to ask. There are several strengths to this study. First, for a qualitative study, it is extensive, with in-depths interviews with 28 women, from different socio-economic backgrounds. Second, whereas most qualitative health research uses an abbreviated grounded theory, in this study the full iterative cycle was performed: after 10 interviews a preliminary data analysis took place, on the basis of which the topic list was improved, the researchers returned to the field and new interviews were undertaken until data saturation was achieved. Third, it is the first such study to be done in the Netherlands, with a maternity system known for its physiological approach to childbirth and its general acceptance among both public and professionals of home birth as a regular option for healthy women with a physiological pregnancy. Fourth, it is part of the larger WONDER study project, from which two literature studies have already been published [2, 5]. Triangulation between the results of literature studies and the data analysis of the interviews has heightened the validity of this research.

Another strength of the study is its critical reflection on validity, by having a feedback focus group discussion with a representative sample of the study population.

Naturally, there are also limitations to this study. First, one could assume that because this study was performed in the specific setting of Dutch maternity care, the results are not necessarily applicable to other countries and healthcare systems. However, the phenomenon of ‘birthing outside the system’ is not specific for the Netherlands, and most of the themes that were found in this research are in accordance with findings from other studies elsewhere. Second, the sampling method can be seen as a limitation. There is no formal registration of women who go against advice in choosing their method and/or place of birth, therefore interviewers had to rely on snowball methods and internet fora. It is possible that participants with activist views on home birth in a high risk pregnancy and UC are over represented. The researchers actively searched for negative cases of women who regretted their choice, but could not find any. Moreover, it seems safe to assume, that for every woman who chooses to go against medical advice, there are likely many who have similar misgivings, but opt, for various reasons, to stay within the system. This should be a focus for future research.

## Conclusion

This qualitative study analyzed the motivations of Dutch women who chose home birth in a high risk pregnancy or unassisted childbirth, against medical advice. Four major themes were found: 1) Discrepancy in the definition of superior knowledge, 2) Need for autonomy and trust in the birth process, 3) Conflict during negotiation of the birth plan, and 4) Search for different care. This study shows that, even though maternity care in the Netherlands has, in comparison to other developed countries, a low rate of interventions and a relatively high home birth rate, some of the themes mentioned by Dutch women as motivation for choosing to go against medical advice are similar to those found in studies elsewhere.

From the data one theme emerged that covered all of the other themes and this was ‘Fear’. This theme refers both to the participants’ fear (of interventions and negative consequences of their choices) and to the providers’ fear (of a bad outcome). Where for some women it was a positive choice, for the majority of women in this study the choice for a home birth in a high risk pregnancy or a UC was a negative one.

Recommendations for maternity caregivers can be summarized as: 1) Rethink risk discourse, 2) Respect a woman’s trust in the birth process and her autonomous choice, 3) Have a flexible approach to negotiating the birth plan using the concept of shared decision making 4) Be aware of alternative delivery care providers and other sources of information used by women, and 5) Provide maternity care without spreading or using fear.

## Abbreviations

BMI: Body mass index
C-section: Cesarean section
CTG: Cardio-tocography
LMWH: LOW Molecular Weight Heparin
MPV: History of Manual Placental Removal
PERISTAT: Perinatal statistics
PPH: Post Partum Hemorrhage > 1000 ml in previous delivery
PROM: Prelabor Rupture of Membranes
UC: Unassisted childbirth
UK: United Kingdom
USA: United States of America
VBAC: Vaginal birth after cesarean
WONDER (study): Why women want Other or No DElivery caRe

## References

[1] Christiaens W, Nieuwenhuijze MJ, de Vries R (2013). Trends in the medicalisation of childbirth in Flanders and the Netherlands. Midwifery.

[2] Hollander M, van Dillen J, Lagro-Janssen T, van Leeuwen E, Duijst W, Vandenbussche F (2016). Women refusing standard obstetric care: maternal-fetal conflict or doctor-patient conflict?. J Preg Child Health.

[3] Cherry A (2007). The detention, confinement, and incarceration of pregnant women for the benefit of fetal health. J Gender & L.

[4] Hickman A (2010). Born (not so) free: legal limits on the practice of unassisted childbirth or freebirthing in the United States. Univ Minn Law Rev.

[5] Holten L, de Miranda E (2016). Women′s motivations for having unassisted childbirth or high-risk home birth: an exploration of the literature on ‘birthing outside the system’. Midwifery.

[6] Feeley C, Thomson G (2016). Why do some women choose to freebirth in the UK? An interpretative phenomenological study. BMC Pregnancy Childbirth.

[7] Plested M, Kirkham M (2016). Risk and fear in the lived experience of birth without a midwife. Midwifery.

[8] Offerhaus PM, Geerts C, de Jonge A, Hukkelhoven CW, Twisk JW, Lagro-Janssen AL (2015). Variation in referrals to secondary obstetrician-led care among primary midwifery care practices in the Netherlands: a nationwide cohort study. BMC Pregnancy Childbirth.

[9] Tong A, Sainsbury P, Craig J (2007). Consolidated criteria for reporting qualitative research (COREQ): a 32-item checklist for interviews and focus groups. Int J Qual Health Care.

[10] Charmaz K (2007). Constructing grounded theory. A practical guide through qualitative analysis.

[11] Peristat. http://www.europeristat.com/reports/national-perinatal-health-reports.html

[12] Scamell M, Alaszewski A (2012). Fateful moments and the categorisation of risk: midwifery practice and the ever-narrowing window of normality during childbirth. Health Risk Society.

[13] Bisits A (2016). Risk in obstetrics-perspectives and reflections. Midwifery.

[14] Martyn C. Risky business: doctors' understanding of statistics. BMJ. 2014;349 10.1136/bmj.g5619 25230984

[15] Pel M, Heres MH, Hart AA, van der Veen F, Treffers PE (1995). Provider-associated factors in obstetric interventions. Eur J Obstet Gynecol Reprod Biol.

[16] Van Wagner V (2016). Risk talk: using evidence without increasing fear. Midwifery.

[17] Chadwick RJ, Foster D (2013). Negotiating risky bodies: childbirth and negotiations of risk. Health Risk Society.

[18] Feeley C, Burns E, Adams E, Thomson G (2015). Why do some women choose to freebirth? A meta-thematic synthesis, part one. Evidence Based Midwifery.

[19] Miller A (2009). Midwife to myself: birth narratives among women choosing unassisted home birth. Sociol Inq.

[20] Lindgren H, Radestad I, Christensson K, Wally-Bystrom K, Hildingsson I (2010). Perceptions of risk and risk management among 735 women who opted for a home birth. Midwifery.

[21] Viisainen K (2001). Negotiating control and meaning: home birth as a self- constructed choice in Finland. Social Sci Med.

[22] Boucher D, Bennett C, McFarlin B, Freeze R (2009). Staying home to give birth: why women in the United States choose home birth. J Midwifery Women’s Health.

[23] Murray-Davis B, McNiven P, McDonald H, Malott A, Elarar L, Hutton E (2012). Why home birth? A qualitative study exploring women's decision making about place of birth in two Canadian provinces. Midwifery.

[24] Freeze R. Born free: Unassisted child birth in North America (Thesis). University of Iowa, written 2008, retrieved April 22 2015 (https://ir.uiowa.edu/cgi/viewcontent.cgi?article=1387&context=etd).

[25] Symon A, Winter C, Donnan P, Kirkham M (2010). Examining autonomy's boundaries: a follow up review of perinatal mortality cases in UK independent midwifery. Birth.

[26] King J, Moulton B (2006). Rethinking informed consent: the case for shared medical decision-making. Am J Law Med.

[27] Nieuwenhuijze MJ, Korstjens I, de Jonge A, de Vries R, Lagro-Janssen A (2014). On speaking terms: a Delphi study on shared decision-making in maternity care. BMC Pregnancy Childbirth.

[28] Cameron H. Expert on her own body: contested framings of risk and expertise in discourses on unassisted childbirth (Thesis). Lakehead University, Retrieved April22 2015 https://knowledgecommons.lakeheadu.ca/xmlui/handle/2453/526

[29] Wickham S (2008). Unassisted birth: listening and learning from the minority. Practising Midwife.

[30] Symon A, Williams B, Adelasoye QA, Cheyne H (2015). Nocebo and the potential harm of 'high risk' labelling: a scoping review. J Adv Nurs.

[31] Jefford E, Jomeen J (2015). “Midwifery abdication”: a finding from an interpretive study. Int J Childbirth.

[32] Healy S, Humphreys E, Kennedy C (2016). Midwives' and obstetricians' perceptions of risk and its impact on clinical practice and decision-making in labour: an integrative review. Women Birth.

[33] Henshall C, Taylor B, Kenyon S (2016). A systematic review to examine the evidence regarding discussions by midwives, with women, around their options for where to give birth. BMC Pregnancy Childbirth.

[34] Lothian J (2006). Birth plans: the good, the bad, and the future. J Obstet Gynecol Neonatal Nurs.

[35] Jenkinson B, Kruske S, Stapleton H, Beckmann M, Reynolds M, Kildea S (2015). Maternity care plans: a retrospective review of a process aiming to support women who decline standard care. Women and Birth.

[36] Mei JY, Afshar Y, Gregory KD, Kilpatrick SJ, Esakoff TF (2016). Birth plans: what matters for birth experience satisfaction. Birth.

[37] DeBaets AM (2017). From birth plan to birth partnership: enhancing communication in childbirth. Am J Obstet Gynecol.

[38] Keedle H, Schmied V, Burns E, Dahlen HG (2015). Women’s reasons for, and experience of, choosing a home birth following a caesarean sction. BMC Pregnancy Childbirth.

[39] Gaskin IM. Spiritual midwifery 4th revised ed. 2002. Book Company Publishing.

[40] Gaskin IM. Ina May’s Guide to Childbirth. 2008. Ebury Publishing.

[41] Shanley LK. Unassisted Childbirth. 3rd Ed. 2016. Bergin & Garvey.

[42] Vadeboncoeur H. Birthing normally after a caesarean or two. 2011. Fresh Heart Publishing.

[43] Dahlen HG, Jackson M, Stevens J (2011). Home birth, freebirth and doulas: casualties and consequences of a broken system. Women Birth.

[44] Green JM, Renfrew MJ, Curtis PA (2000). Continuity of carer: what matters to women? A review of the evidence. Midwifery.

[45] Cheyney M (2008). Home birth as systems-challenging praxis: knowledge, power, and intimacy in the birthplace. Qual Health Res.

[46] Dahlen HG (2016). The politicisation of risk. Midwifery.

[47] Dahlen H (2010). Undone by fear? Deluded by trust?. Midwifery.

[48] Porter ME, Olmsted Teisber E. Redefining Health Care. Boston: Harvard Business Review Press; 2006.

